# Subjective social status and health among older adults in China: the longitudinal mediating role of social trust

**DOI:** 10.1186/s12889-023-15523-z

**Published:** 2023-04-03

**Authors:** Jingjing Zhou, Wei Guo, Hang Ren

**Affiliations:** 1 School of Sociology and Population Studies, Nanjing University of Posts and Telecommunications, Jiangsu Province, Nanjing, 210023 People’s Republic of China; 2 Department of Social Work and Social Policy, School of Social and Behavioral Sciences, Nanjing University, Jiangsu Province, Nanjing, 210023 People’s Republic of China; 3 The Centre for Asia-Pacific Development Studies, Nanjing University, Jiangsu Province, Nanjing, 210023 People’s Republic of China; 4 Institute of Population Studies, Nanjing University of Posts and Telecommunications, Nanjing, 210042 People’s Republic of China

**Keywords:** Subjective social status, Social trust, Self-rated health, Older adults, China

## Abstract

**Background:**

From a developmental perspective, this study explored the interplays between subjective social status (SSS), social trust (ST), and health status measured by self-rated health (SRH) among older adults in the context of China. It also tested the longitudinal mediation of ST between SSS and SRH.

**Methods:**

After excluding samples with missing values, we analyzed 4,877 individual responses from those aged 60 years or older, extracted from the China Family Panel Studies (CFPS) data in 2014, 2016, and 2018. We used latent growth modeling to test the hypothesized relationships among their SSS, ST, and SRH.

**Results:**

Latent growth modeling based on bootstrapping showed that the SSS, ST, and SRH of older adults all increased linearly and that the mechanism of SSS acted on the SRH as follows: the initial level of SSS indirectly influenced the initial level and the growth rate of the SRH, respectively, through the initial level of ST, and the initial level and growth rate of SSS played an indirect role in the growth rate of the SRH through the growth rate of ST.

**Conclusion:**

The findings have practical implications for promoting health for older adults and realizing active aging in China. Therefore, we recommend establishing a family-centered and community-supported social support system for those with lower social status among older adults and a friendly community environment with various social, cultural, and recreational activities to improve the ST among older adults, thereby improving their health.

## Background

Influenced by such factors as increasing life expectancy and decreasing fertility rate, the growth rate and scale of China’s population aged 60 and above exceeds that of most developed countries [1]. Census data released by China’s National Bureau of Statistics shows that from the fifth census in 2000 to the seventh census in 2020, China’s population aged 60 and above increased from 126 to 264 million, accounting for 18.7% of the total, up from 10.2%. As the country with the largest number of older adults in the world, China is in the transition stage from mild aging to moderate aging, and such changes have far-reaching impacts on its economic and social development [2].

Entering old age and changes in physiology, socioeconomic status, and social roles, older adults see their health status deteriorating, making them the largest “health-vulnerable” group in China [3]. Additionally, the COVID-19 pandemic increased their health risks and contributed to negative social emotions [4]. Among the many factors affecting their health, social class has been the focus of scholarly attention in recent years. Unequal standing in the social structure affects one’s health, resulting in a health gradient [5]. In China, with the transition from the traditional family structure to the nuclear family model, a series of changes have occurred in the intergenerational family relationship. In terms of power relations, older adults no longer enjoy the authority to dominate their offspring; in terms of intergenerational exchange, the moral rules fade in their role of obliging the offspring to support older adults. In addition, social competition and status anxiety caused by class differentiation are being transferred to older adults through intergenerational division of labor and intergenerational exploitation within the family [6]. All these changes have resulted in the instability of the subjective and objective social statuses of older adults and affected their physical and mental health [7].

Moreover, research has confirmed the effect of social trust on health status in many ways. For instance, social trust (ST), which varies by social status, intensifies health disparities in resource acquisition [8]. Subjective social status (SSS) reflects resources corresponding to social status [9], i.e., the more social resources one has, the higher their ST level [10]. In addition, SSS affects ST, which in turn affects health, raising the possibility that ST mediates the linkage between SSS and health [11]. In addition, SSS, ST, and the self-rated health (SRH) of older adults change over time [12]. However, few studies have probed the relationships between SSS, ST, and health status of older adults from a developmental perspective, not least in the context of China. Thus, this study used the China Family Panel Studies (CFPS) data in 2014, 2016, and 2018 to explore the relationships between SSS, ST, and health status of older adults in China from a developmental point of view. In addition, we tested the longitudinal mediating effect of ST on the action of SSS on health to inform policies for promoting health and active aging of older adults.

### Literature review

#### SSS and health

In recent studies on people's health, social status has been a focal point. According to the theory of relative deprivation, people can easily recognize their relative status. Once they think they are at a low social status, depression will occur, which will adversely affect their mental health [13]. Some scholars have provided evidence that good working and living environments, higher education, higher income, and so on usually have direct effects on individual health [14]. Meanwhile, other scholars have discussed the indirect effects of social status such as its impact on individual health through its influence on the accessibility and quality of medical services, scientificity of dietary structure, and mental health status [15].

In addition to objective social status, an increasing number of studies since 2000 have shown that the effect of SSS on health is far greater than that of objective social status [16]. SSS is a person’s perception of his/her own position in the social hierarchy; it describes how one perceives his/her socioeconomic conditions relative to others and the social stratum to which he/she belongs [12]. Individuals’ subjective evaluation of their social status has a direct effect on their self-rated health (SRH) results. In fact, an individual's subjective assessment of his or her social status is a better predictor of his or her health status and its changes [17] and even a reliable tool for predicting mortality in older adults [18, 19]. Furthermore, compared with objective social status indicators, SSS more importantly affects health, and its correlation with physical and mental health elements, such as anti-stress cortisol, metabolic stress response, common cold susceptibility, and depression, is consistent and stable [20–22]. A large number of empirical studies argued that low SSS is accompanied by psychological pressure and negative perception such as identity anxiety, negative emotion and shame, which have a negative impact on mental health [23–25]. In addition to subjective status indicators, "perceived class mobility," which is closely related to subjective status, also significantly affects SRH. For example, Wu and Chen examined the relationships between SSS, perceived class mobility, and health, and they found that compared with rural areas where social mobility is low, for urban residents, perceived class mobility has a significant positive effect on their SRH [26]. After individuals move to a city, their subjective judgment about fairness, rejection, daily life environment, and overall adaptation to urban life significantly affect their self-evaluation of health. In general, in prior research on the impact of SSS on health, some scholars based their research on cross-section data, while a few of others used tracking data to predict the relationship between SSS and health [17]. For instance, a study tracking the health-related quality of life among old folks in Hong Kong found that the higher the SSS, the healthier the quality of life [27].

#### SSS and ST

Many studies have demonstrated the positive predictive effect of objective social status on ST from the angle of the "resource-based theory". The amount of disposable resources of individuals varies among different social classes. The more resources an individual possesses, the more resistant and the more resilient he/she is to risks from interactions with strangers. Delhey and Newton found that the more resources and wealth individuals possess, the more likely they are to trust others and the higher their level of ST [28]. Hence, an individual at a higher social class is more able to withstand the potential loss of trusting strangers; that is, the higher the individual's objective social status, the higher the level of trust is [29]. For instance, Delhey and Newton used questionnaires to measure ST and concluded that the resources and wealth possessed by individuals are significantly, positively correlated with their level of trust [28]. Based on the tracking data of CFPS in 2018, Li and Jiang established a theoretical model of social status and social choice and found that social status positively affected social trust [30].

Compared with objective social status, an individual's SSS, as a subjective perception of their objective socioeconomic conditions, may have a stronger relationship with or higher predictability on social trust [31]. Studies have found that SSS is more related to some specific indicators of an individual's perception of the present and future, such as sense of control and subjective well-being [32, 33]. In other words, individuals' subjective evaluation of their own social status has a significant positive impact on their happiness and ST [31]. Higher subjective status portend more resources and confidence, which lifts the "threshold to disaster" and prospective level of trust [34]. In addition to the positive effect of SSS on ST, studies have also demonstrated the relationship between SSS and social distrust. For example, Yu et al. based on the relative deprivation and equity theory, explored the relationship between SSS and social distrust of Chinese college students and found that a lower SSS indicates a higher level of distrust, and that an individual’s sense of relative deprivation plays a mediating role between SSS and social distrust [35].

#### ST and Health

Social trust plays a pivotal role in human social life. It is not only an important way for individuals to reduce psychological complexity and obtain ontological security, but also an effective facilitator of social integration to maintain social order and promote social harmony and stability [36]. Scholars have illustrated from different perspectives that the higher the level of ST, the higher the level of mental health [37–39]. Some scholars have demonstrated that from the perspective of social participation. Among them, Rostila believes that ST makes individuals more willing to participate in social activities by exerting internalities, resulting in higher SRH [40]. Some analyzed the positive effects of ST on health from the perspective of social capital. Berkman and Syme found in a long-term follow-up study of mortality in Alameda County, California that people with less social connections were two to three times more likely to die than those with more social connections [41]. In other words, an individual’s social capital enables the person to obtain more health knowledge and information through the establishment of social network, thus to improve the ability of self-defense and reduce risks of falling ill.

In addition, some scholars have revealed from a more macroscopic perspective that ST, as a form of policy intervention, is of great significance to the improvement of the health level of older adults [42]. The government provides medical and pension services for older adults to not only improve their health level, but also create an atmosphere of ST in the macro social environment, so that older adults have trust in the government [43]. A follow-up study conducted on the grassroots ST among older adults in certain neighborhoods found that ST effectively reduced the risk of diseases such as cardiovascular disease and Alzheimer's among the older adults [44] and that in neighborhoods with a high level of ST the older adults had increased ability to resist the risk of disease due to a high degree of reciprocity with their respective neighborhood [45, 46].

#### ST as mediator

ST refers to the anticipations of members of society regarding normal, honest and cooperative behaviors based on shared social norms [47]. The higher one’s SSS, the stronger their sense of ST [48]. Meanwhile, people with high ST level tend to be healthier [49]. SSS influences trust because of its effect on people’s relative vulnerability. If stability is not guaranteed, a lack of self-security makes the risk of trusting others more salient [50]. Some scholars used regression models to analyze the differences in ST among older adults with different social status, and found that among older adults when their social status is low, their ST would decline, whereas the ST of those with higher social status would rise [51]. Social trust can promote the health and well-being of older adults by reducing negative emotions. The higher the level of ST, the more significantly it acts on the mental and physical health of older adults [52, 53].

Although related studies have respectively probed the impact of SSS on health, they have rarely studied or explained in a well-rounded and specific manner the impact of ST on health and how SSS affects ST among older adults [54], and the interaction between SSS, ST, and health among older adults. A handful of studies have put forward from the vantage point of social capital that older adults with higher objective social standing obtain more social capital and associated health advantages [55, 56]. They used theoretical models to reveal that social capital is a possible path by which objective social status affects mental health. For instance, with the stress process model, it is discussed that different stressors arise from different objective social statuses and that a lower objective social status heightens the risk of psychological distress, causing damage to mental health. In contrast, social capital, including social trust and social support, reduces psychological pressure [57]. Some scholars reasoned from follow-up data that self-rated family social status in childhood has a negative predictive effect on the degree of depression in adulthood, that ST plays a mediating role therein for middle-aged and older adults, and that a high ST level offsets the adverse effects of self-rated low family social status in early years on depression in later life [58].

#### Limitations of prior studies and hypothesis development

The basis of most studies in the literature review is cross-sectional data. Consequently, researchers only looked into the direct action of SSS and ST on health [18] without analyzing these variables’ trends. Moreover, the ST and SRH of older adults may change over time [44, 59], highlighting the need to explore whether ST’s initial level or growth rate has a longitudinal mediating role in linking SSS and health. Referencing studies that have used latent growth modeling (LGM) for mediation analysis [60], this study explored the trajectory of ST to test the longitudinal mediation of its initial level and growth rate on the association between SSS and health. The core hypotheses of this study are as follows:

- **H**_**1**_**:** The initial level and growth rate of SSS directly affect the initial level and growth rate of health among older adults.
- **H**_**2**_**:** The initial level and growth rate of SSS influence the initial level and growth rate of health by affecting the initial level and growth rate of ST among older adults.

## Methods

### Data

This study used follow-up data of three time points in 2014, 2016, and 2018 of Peking University’s CFPS. It included only panel data from individuals who provided data in all three waves. The object of study is older adults, so only respondents aged 60 and above entered the analysis. In the process of sampling for follow-up studies, it is inevitable that respondents die, relocate with families, separate from families, or refuse to be followed up [61, 62]. We started with 8,757 older participants interviewed in 2014, among whom 1,562 failed to be followed up in the 2016 survey and an additional 2,318 failed to be followed up in the 2018 survey. Due to varying degrees of data missing on relevant variables in each wave, to ensure the continuity of data and consistency of sample size in the three waves, we used the multiple imputation [63, 64] method to process missing data, so that the data gap was filled with 341 effective samples. Compared with other imputation methods, multiple imputation method takes into account both the variation of the data itself and the variability brought by the imputation process. Since it uses the information of outcome variables, multiple imputation method can also be used in the case of difference error models as opposed to the regression calibration method [65]. Overall, 4,877 individuals contributed the data over the three years in the current study.

### Measures 3

#### Dependent variable

“Health status” was measured by SRH herein [66]. The CFPS questionnaire asked respondents, “How would you rate your health state?” on a five-point scale (ranging from "1 = *poor*" to "5 = *excellent"*).

#### Independent variable

The measurement of SSS reflects one’s objective situation and accounts for the subjective criteria of the individual [67, 68]. The CFPS assessed SSS by asking respondents, “What is your social status locally?” The respondents answered the question according to their situation on a five-point scale from 1 (*very low*) to 5 (*very high*).

#### Mediating variable

There are many classifications of ST from various perspectives, such as the object of trust, position in the pattern of difference sequence, and institutional characteristics. Some examples include generalized and particularized trust, individual and organizational trust, intra-group and out-group trust, and institutionalized and non-institutionalized trust [69]. CFPS evaluated trust from the perspective of generalized trust. Respondents answered, “Generally speaking, do you think most people are trustworthy or the more careful, the better in dealing with others?” The level of trust ranged from 1 (*the more careful, the better*) to 5 (*most people are trustworthy*).

#### Covariates

The covariates herein were composed of factors relating to social background and individual factors. Hukou (registered residence) is an important social background factor in this study. In China's *hukou* system, *hukou* is divided into agricultural and urban types (0 = rural, 1 = urban). Although beginning in 2005, the Chinese government has set out to reform the system, the urban older adults are still superior to a large extent to the rural ones in terms of social security, health care resources and so on, which has directly caused the difference in health between the two groups of people [70]. In addition, whether the registered residence is where one currently lives, i.e., whether one is migrant, will directly affect the reimbursement rate of various types of social security [71]. Therefore, in addition to hukou, one’s migrant status (0 = migrant, 1 = non-migrant) was included as a covariate.

Previous studies have shown that years of work experience and a higher level of education may increase the likelihood of joining the Communist Party of China (CPC), thereby gaining more political and social capital, higher wage income, and positively affecting one’s SSS [72]. Therefore, the CPC membership (0 = no, 1 = yes) was also included as one of the covariates.

SSS reflects the perception of one’s objective social status to some extent, and objective social status has been suggested to have an impact on individual health by many studies. Therefore, the measures of objective social status were included in this study, including household income, education level (years of education), and occupational economic status [73]. In the CFPS database, the household income refers to the disposable income of a family, which is derived from the total income minus the total expenditure of the family. The total income of a family comprises business income, wage income, property income, government subsidies or economic support from others, etc. The total expenditure of a family comprises daily expenses such as food, clothing, housing and transportation, education, medical care, culture and leisure, and favors and gifts given to others. For statistical convenience, households with various amounts of disposable income were grouped according to the 2018 China Time Use Survey released by the National Bureau of Statistics of China on January 25. Therefore, households with an annual income less than RMB24,000 formed the low-income group, those with an annual income of RMB24,000–60,000 the lower-middle-income group, those with an annual income of RMB60,000–120,000 the upper-middle-income group, and those with an annual income of RMB120,000 and above the high-income group. In this study, the low-income group, the lower-middle-income group, the upper-middle-income group and the high-income group were assigned a value of 1–4, respectively, in which the higher the value, the higher the disposable income of the family. The occupational economic status referred to the ISEI (International Socioeconomic Index), which measures the occupational status as a weighted average of the practitioner’s education and income, and the higher one’s socioeconomic status, the higher the ISEI [74].

In addition to the abovementioned social background factors, the participation in medical insurance (0 = no, 1 = yes) and the region of the country where one is located (with the east as the control group and the central and western regions as dummy variables) were included as covariates. The covariates relating to individual factors included gender (0 = male, 1 = female), ethnic group (0 = ethnic minority, 1 = Han), age, marital status (0 = single, 1 = married), whether to suffer chronic diseases (0 = no, 1 = yes), independent living ability and depression status. CFPS database refers to the Activities of Daily Living (ADLs) and the Instrumental Activities of Daily Living (IADLs) to measure one’s independent living ability [75, 76], which includes seven indicators, such as "going outdoors independently" and "eating independently". Depression was measured by the Depression Scale with such questions as "how often have you felt excited with nothing in the past month?" and "how often do you feel mentally tense?" The answers to the questions were rated on a five-point scale (1 = almost every day; 2 = often; 3 = half the time; 4 = sometimes; 5 = never).

#### Analysis

SPSS 23.0 was employed to organize data, generate descriptive statistics, and conduct correlation analyses. Before performing the analyses, we used Mplus8.0 to establish a structural equation model between the variables through three steps. First, we used LGM to separately examine the trajectory of each variable by extracting its intercept and slope. The intercept indicated the initial level of the variable, and we fixed all factor loadings at one. The slope represented the growth rate of the variable. We set the factor loadings of the slopes at zero, one, and two to fit a linear trajectory and apply LGM [77]. In the second step, we used SSS and SRH scores from three measurements to establish a conditional LGM to test the direct effect of SSS on the intercept and slope of health. Third, a structural equation model was established between SSS, ST and SRH to measure the direct path between the intercept and the slope of each variable. The common indexes used to test the fitting degree of structural equation models include chi-square (χ2)/df ratio, root mean square error of approximation (RMSEA), comparative fit index (CFI), Tucker-Lewis index (TLI), etc. If the chi-square (χ2)/df ratio is less than 5, RMSEA less than 0.08, CFI and TLI higher than 0.900, a sound model fitting is indicated [78]. In addition, on the basis of the establishment of the model, the bootstrapping process (resampling 1,000 times) was used to verify the significance of the mediating effect. If the 95% confidence interval (CI) does not include 0, the effect will be significant [79].

## Results

### Descriptive statistics and correlation analyses

Table 1 lays out the variables’ baseline sample characteristics and their means and standard deviations over time. The mean age of the sample was 67.62. Women accounted for 46.67%, Han Chinese accounted for 92.54%, and urban residents accounted for 51.65% of the total sample. Other demographics included 96.90% married, 4.82 years (average) of education, and 14.89% members of the CPC. 2.85% of the sample was migrants, 94.30% had medical insurance, and 26.80% suffered from chronic diseases. The sample’s mean value of the independent living ability index was 0.93, mean value of the depression level was 1.68, and 52.78% of the sample came from the central and western regions of China. Their ISEI showed a decreasing trend over time, and their AHI, SSS, SRH, and ST levels showed an increasing trend.

**Table 1:** Sample characteristics at baseline and means and standard deviations of variables over time

Variables	Time 1		Time 2		Time 3		Time 4	
	**M (%)**	**SD (Range)**	**M**	**SD**	**M**	**SD**	**M**	**SD**
Age	67.62	5.85						
Female	(46.67)	(0–1)						
Han Chinese	(92.54)	(0–1)						
Non-Agricultural	(51.65)	(0–1)						
Married	(96.90)	(0–1)						
Education	(4.82)	4.66						
CPC membership	(14.89)	(0–1)						
Migrant status	(2.85)	(0–1)						
Medical insurance	(94.30)	(0–1)						
Chronic diseases	(26.80)	(0–1)						
Independent living ability	0.93	0.17						
Depression	1.68	0.55						
Central China	(32.13)	(0–1)						
Western China	(20.65)	(0–1)						
Low-income group	(83.62)	(0–1)						
Lower-middle-income group	(12.73)	(0–1)						
Upper-middle-income group	(2.75)	(0–1)						
High-income group	(0.90)	(0–1)						
ISEI	26.27	9.52	26.68	12.65	25.85	11.81	26.28	11..63
SSS	3.26	1.29	3.05	1.26	3.25	1.29	3.49	1.30
SRH	2.71	1.33	2.58	1.35	2.72	1.33	2.83	1.30
ST	3.52	1.91	3.13	1.99	3.53	1.92	3.90	1.74

*N* = 4,877. *CPC* Communist Party of China, *ISEI* International Socioeconomic Index, *SSS* Subjective social status, *SRH* Self-rated health, *ST* Social trust

Table 2 displays each variable’s mean, standard deviation, and the correlation among key variables in the three measurements (Time 1, 2, and 3). Significant positive correlation existed between SSS, ST, and SRH one another; the coefficients between the variables were all less than 0.8; and the data did not suffer from significant multicollinearity, meeting the prerequisites for the mediation test [80].

**Table 2:** Correlation analyses between key variables

	**1**	**2**	**3**	**4**	**5**	**6**	**7**	**8**	**9**
T1 SSS	1								
T2 SSS	0.57**	1							
T3 SSS	0.38**	0.37**	1						
T1 ST	0.36**	0.28**	0.20**	1					
T2 ST	0.23**	0.23**	0.20**	0.46**	1				
T3 ST	0.11**	0.11**	0.18**	0.37**	0.48**	1			
T1 SRH	0.19**	0.13**	0.10**	0.16**	0.15**	0.15**	1		
T2 SRH	0.15**	0.16**	0.14**	0.14**	0.17**	0.15**	0.55**	1	
T3 SRH	0.11**	0.11**	0.14**	0.10**	0.15**	0.18**	0.45**	0.49**	1
Mean	3.05	3.25	3.49	2.58	2.72	2.83	3.13	3.53	3.90
SD	1.26	1.29	1.30	1.35	1.33	1.30	1.99	1.92	1.74

^**^ *p* < 0.01. *SSS* Subjective social status, *ST* Social trust, *SRH* Self-rated health, *SD* Standard deviation; T1, T2, and T3 represent 2014, 2016, and 2018, respectively; 1–9 numbers in the header represent T1 SSS, T2 SSS, T3 SSS, T1 ST, T2 ST, T3 ST, T1 SRH, T2 SRH, and T3 SRH, respectively

### Trajectories of SSS, ST, and SRH

We used unconditional LGMs to explore the trajectories of the studied variables and individually fit the SSS, ST, and SRH scores. Table 3 describes the fit indicators of the three models and the means of the intercepts and slopes. The study found that the models of SSS, ST, and SRH were well fitted, and the mean slope was positive, indicating that the development of the SSS, ST, and SRH all showed an increasing linear trend. However, the partial correlation coefficients were negative, indicating that the higher the initial level of the SSS, ST, and SRH, the slower the growth rate [81].

### Direct effect of SSS on the initial level and growth rate of SRH

**Table 3:** LGM fitting indicators, intercepts, and slopes of key variables

Variables	χ^2^	df	RMSEA	CFI	TLI	Mean		Partial Correlation
						**Intercept**	**Slope**	
SSS	29.316	17	0.012	0.996	0.987	0.976***	0.988***	-0.712***
ST	23.003	17	0.009	0.998	0.994	0.940***	0.973***	-0.494***
SRH	58.784	17	0.022	0.991	0.973	0.802***	0.934***	-0.554***

^***^ *p* < 0.001. SSS = subjective social status, *ST* Social trust, *SRH* Self-rated health

**Fig. 1:**
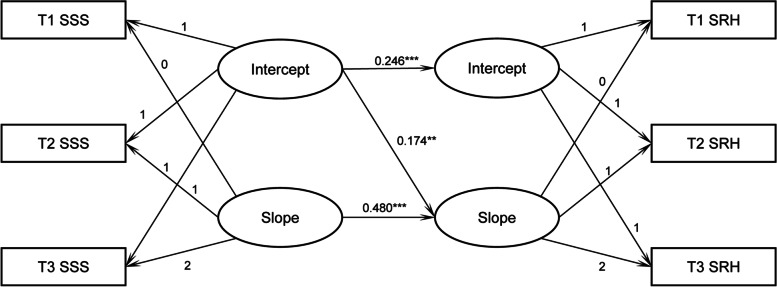
Conditional LGM with SSS as independent variable. *p < 0.05, ***p < 0.001. SSS = subjective social status, SRH = self-rated health; T1, T2, and T3, respectively, signifies 2014, 2016, and 2018

We constructed a conditional LGM, with SSS as the independent variable and SRH as the dependent variable, to examine whether SSS can affect the initial level and growth rate of SRH. Figure 1 shows abbreviated results of the path analysis in this model as the standardized path coefficients of the key variables. The conditional model had a good fit (χ^2^/*df* = 224.191/72, CFI = 0.980, TLI = 0.970, RMSEA = 0.021). The SSS intercept significantly and positively influenced the SRH intercept (*ß* = 0.246*, p* < 0.001) and slope (*ß* = 0.174*, p* < 0.01), indicating that the higher the initial level of SSS, the higher the initial level of the SRH, and the faster the growth rate of the SRH. The SSS slope significantly and positively affected the SRH slope (*ß* = 0.480, *p* < 0.001), indicating that the faster the improvement of SSS among older adults, the faster the improvement of their health.

### Longitudinal mediation of ST between SSS and SRH

#### Model establishment and fitting

**Fig. 2:**
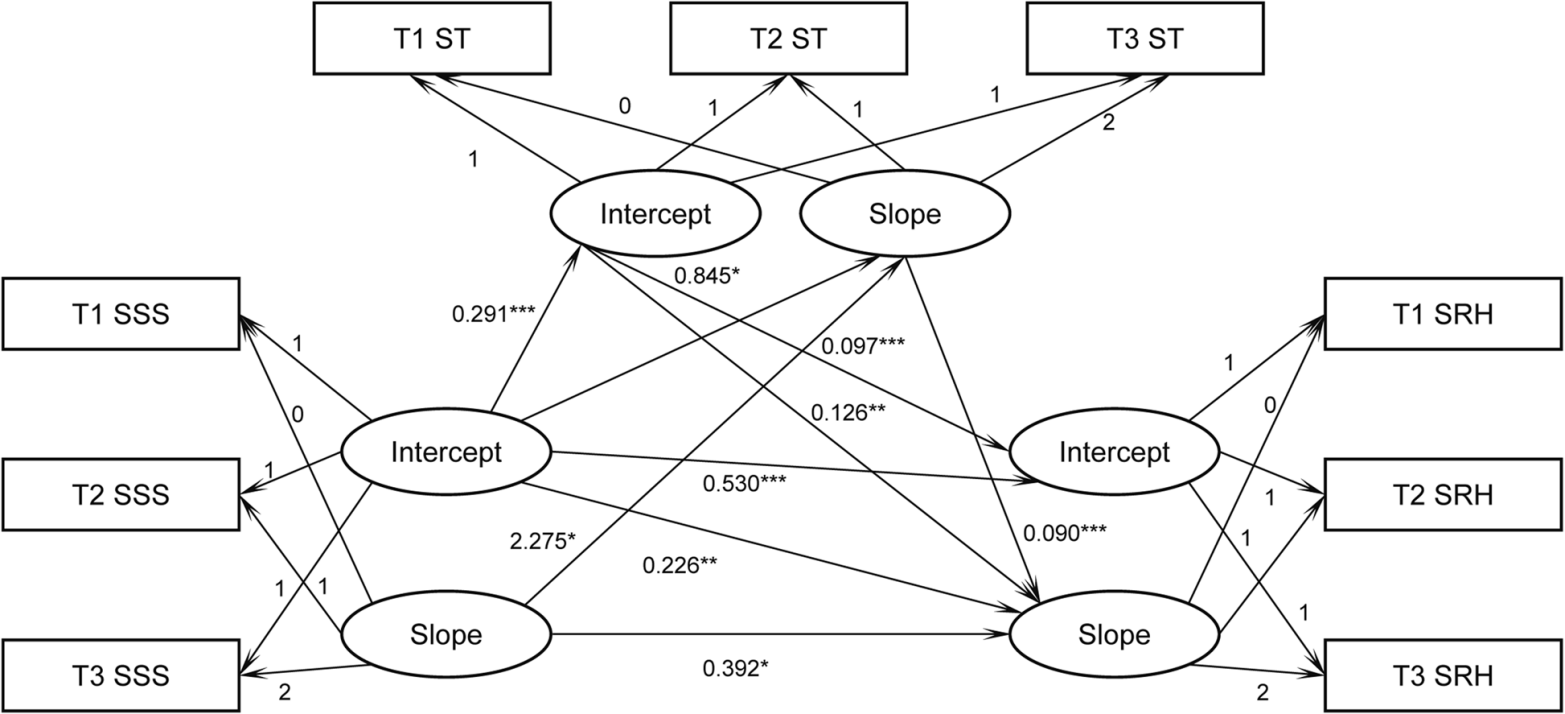
Longitudinal mediation model with ST as mediator. **p < 0.01, ***p < 0.001. SSS = subjective social status, SRH = self-rated health; T1, T2, and T3, respectively, signifies 2014, 2016, and 2018

We used the LGMs of SSS, ST, and SRH to examine the longitudinal mediating role of ST. We tested the mediation of ST by establishing a structural equation model whose fit indicators were also tested. The results revealed that the model had a good fit (χ^2^/*df* = 343.980/102, CFI = 0.979, TLI = 0.963, RMSEA = 0.022). Figure 2 illustrates the final model.

#### Direct effects between the variables in the longitudinal mediation model

Table 4 describes the direct paths between the SSS, ST, and SRH. When we controlled the covariates, including gender, age, ethnicity, hukou, education, CPC membership, AHI, ISEI, participation in medical insurance, whether to suffer chronic diseases, independent living ability, depression status, and region of the country where one is located, the intercept of SSS had a significant positive effect on the intercept of ST (*ß* = 0.291, *p* < 0.001) and the slope of ST (*ß* = 0.845*, p* < 0.05). This result indicates that the initial level of SSS significantly and positively affected the initial ST level and promoted the ST growth rate. In addition, the slope of SSS significantly and positively impacted the slope of ST (*ß* = 2.275*, p* < 0.05), indicating that the growth rate of SSS positively contributed to the growth rate of ST. Furthermore, the intercept of SSS had a significant positive effect on the intercept of SRH (*ß* = 0.530, *p* < 0.001)). It also significantly and positively predicated the SRH slope (*ß* = 0.226, *p* < 0.01), signifying that the higher the SSS of older adults, the higher the initial level and the faster the growth rate of their health. The intercept of ST also significantly and positively predicated the intercept and slope of SRH (*ß* = 0.097, *p* < 0.001; *ß* = 0.126, *p* < 0.01), indicating that the initial level of ST had a significant and positive effect on the initial level and growth rate of health. Finally, the SSS slope (*ß* = 0.392, *p* < 0.05) and ST slope (*ß* = 0.090, *p* < 0.001) significantly and positively affected the SRH slope, indicating the SSS and ST growth rates promoted the SRH growth rate.

**Table 4:** Direct paths in the longitudinal mediation model

Path coefficient			*ß*	*B*	S.E	C.R	*p*
SSS intercept	→	ST intercept	0.291	0.260	0.018	16.245	0.000
SSS intercept	→	ST slope	0.845	0.069	0.336	2.515	0.012
SSS slope	→	ST slope	2.275	0.587	1.021	2.229	0.026
SSS intercept	→	SRH intercept	0.530	0.716	0.021	25.340	0.000
ST intercept	→	SRH intercept	0.097	0.147	0.023	4.242	0.000
SSS intercept	→	SRH slope	0.226	0.126	0.084	2.690	0.007
SSS slope	→	SRH slope	0.392	0.673	0.180	2.171	0.030
ST intercept	→	SRH slope	0.126	0.077	0.037	3.405	0.001
ST slope	→	SRH slope	0.090	0.600	0.019	4.737	0.000

*B* = unstandardized coefficient, *ß* = standardized coefficient, *SE* Standard error, *CR* Critical ratio, *SSS* subjective social status, *ST* Social trust, *SRH* Self-rated health

#### Test of the longitudinal mediation effect of ST

We used bootstrapping (1,000 re-samples) to verify the mediating effect of the intercept and slope of ST, respectively, to explore the longitudinal effect of ST in the association between SSS and SRH. The model included four indirect paths: (1) SSS intercept →ST intercept →SRH intercept, (2) SSS intercept →ST intercept →SRH slope, (3) SSS intercept →ST slope →SRH slope, and (4) SSS slope →ST slope →SRH slope. The bootstrapping results showed that the four indirect paths were significant (see Table 5), indicating that SSS indirectly affected the health development of older adults. In addition, ST’s initial level and growth rate played a longitudinal mediating role between SSS and health. Among them, the ST intercept partially mediated the interplay between SSS and SRH intercepts, accounting for 5.01% of the total effect; the ST intercept partially mediated the linkage between the SSS intercept and SRH slope, accounting for 10.9% of the total effect; the ST slope partially mediated the interaction between the SSS intercept and SRH slope, accounting for 22.4% of the total effect; and the ST slope partially mediated the connection between the SSS and SRH slopes, accounting for 34.4% of the total effect.

**Table 5:** Indirect paths in the longitudinal mediation model

Indirect Paths	Indirect Effect	*p*	95% Bootstrap		Proportion of Mediating Effect
SSS intercept → ST intercept →SRH intercept	0.028	0.000	0.016	0.042	5.01%
SSS intercept → ST intercept →SRH slope	0.037	0.001	0.019	0.063	10.9%
SSS intercept →ST slope →SRH slope	0.076	0.003	0.007	0.315	22.4%
SSS slope →ST slope →SRH slope	0.205	0.011	0.049	0.741	34.4%

*SSS* Subjective social status, *ST* Social trust, *SRH *Self-rated health

## Discussion

For the development trends of older adults’ SSS, ST, and health, we used LGM analysis to come by the following findings. First, older adults’ SSS increased steadily from 2014 to 2018, but the higher the initial level, the slower the growth rate, echoing the results of previous studies. Since 2017, the Chinese government has developed a multi-dimensional policy system and framework encompassing elderly care, health care, social security, social participation, rights and interests protection, etc., so that the subjective and objective social statuses of older adults have been greatly improved [48]. However, as older adults enter the later stage of life, their income, occupation, and living status tend to be stable, hence a slower increase in their SSS [82]. Second, the ST of older adults varied significantly from 2014 to 2018, but a higher initial level corresponded to a slower growth rate, which is supported by some literatures from the perspective of social environment, notwithstanding that few studies have analyzed the changes of ST among older adults in China. Since 2012, the Chinese government has taken a multi-pronged approach involving wealth reserve, labor supply, elderly service and product supply systems, technological innovation capability building, social environment construction, etc. to build a more harmonious policy system and society to provide care and respect for older adults [83], which has improved the ST of older adults. However, ST is an important social capital, and its growth is subject to multiple factors at the individual and social levels [84]. For instance, there are limits on ST’s growth rate after reaching a certain level. Finally, the health level of older adults continued to increase from 2014 to 2018, and the higher the initial level, the slower the growth rate, which is consistent with the results of relevant papers on the health status and its trends among older adults in China. Since 2010, the SRH of Chinese urban and rural elderly has steadily improved [70], though their physical health status has gradually declined with aging. But that doesn't mean SRH must decline with aging for older adults. SRH is a comprehensive perception of one’s overall health status [85], which can be influenced by factors other than physiological health. Studies have noted that the SRH of older adults in China does not necessarily decrease with aging mainly for two reasons. On the one hand, the older the elderly, the less demanding they are on themselves and the environment, the less pressure and responsibility they face from family and society, hence the higher levels of mental health [86]. On the other hand, China's profound tradition of "filial piety" lays great stress on children's respect and duty for the elders, so the longevous will receive special support from the government and families, which also improve their SRH [87]. Notwithstanding, a high initial level of SRH does not imply a sustained high growth of it in a given period [88]. Factors such as family support, exercise, access to physical examination, and chronic disease all affect whether the improvement of SRH will sustain for older adults [89].

In this study, SSS directly affected the development trends of the health status of older adults, which supported H_1_. On the one hand, the initial level of SSS significantly and positively affected the initial level and growth rate of SRH, echoing previous research results, which indicate a positive correlation between SSS and health status among older adults. Compared with the objective social status, SSS can more stably predict one’s health status and changes [90]. On the other hand, the faster the improvement of older adults’ SSS, the faster the improvement of their health. As mentioned in relevant studies, within any objective social class, an individual’s health-related quality of life increases with SSS improvement [91].

Before further investigating the longitudinal mediating role of ST between SSS and the health development of older adults, this study examined nine possible direct paths between the trajectories of SSS, ST, and SRH. The results showed that the initial level and growth rate of SSS significantly and positively promoted ST’s initial level and growth rate, which coincides with previous research findings. The higher one’s SSS, the higher their self-perceived indicators such as a sense of control and well-being, hence a higher level of ST [92]. ST’s initial level and growth rate positively affected the initial level and growth rate of health among older adults. As argued in a number of literatures, ST is an important factor influencing the SRH and mental health among older adults, and the higher the level of ST, the more conducive it is to their mental and physical health [52]. Moreover, some scholars have used binary regression models and decision tree classifiers to find a negative relationship between ST and SRH of older adults for whom the lower the level of interpersonal trust, the worse the SRH [93]. This conclusion proposes conversely that there may be a negative correlation between ST and mental health of older adults; that is, low levels of ST may lead to low levels of health.

On the basis that all direct paths in the longitudinal mediation model were significant, this study further tested the indirect paths in the model using the bootstrap method. As a result, we found four indirect paths, which indicated that in addition to the direct influence, ST played a bridge role between SSS and the health of older adults, thereby supporting H_2_ in this study, i.e., sound SSS of older adults indirectly positively affects their mental health level and development. Meanwhile, ST’s initial level and growth rate played a positive longitudinal mediating role between SSS and the health of older adults. This finding is an innovation of the present study by constructing a new framework to explain the relationship between SSS, ST and the health among older adults. The possible explanation is that SSS is the perception and evaluation of objective social status [12], and the improvement of SSS entails the expansion of social networks, the reduction of ST cost, and the ability to carry out better social interactions. The improvement of ST can further bring up the health level of the older adults [94].

This study is not, however, without its limitations. First, the limited tracking times in the CFPS cited herein made it impossible to plot the possible quadratic growth. Future research should track these variables multiple times and use LGM to determine their quadratic growth trajectories [95]. Second, in addition to social trust, other variables may mediate the relationship between SSS and the health of older adults, such as social capital, social support, social participation, and social cognition [96, 97]. Third, in addition to the action of SSS on health, health also exerts influence on SSS [98, 99]. Moreover, some studies using panel data show that changes in SSS are not associated with changes in health [100]. Therefore, more extensive follow-up data can be used to further explore the multi-faceted correlation between SSS and health against different theoretical backgrounds. Fourth, due to data limitations, the health status measurement of the sample did not include objective physical indicators. In future studies, researchers should collect SRH and objective physical check-up indicators to ensure the comprehensiveness of research data. Finally, during the COVID-19 pandemic, besides the spread of the virus threatening people’s health, the social response, such as routinized governance measures, has profoundly affected the SSS, ST, and health status among older adults. However, we could not analyze this impact in-depth due to data limitations. In the future when first-hand survey data is available, researchers should explore the pandemic’s impact on older adults in terms of the mental and physical health [101].

## Conclusion

The study showed that during the study period, the development of SSS, ST, and SRH of older adults generally showed an increasing linear trend. For instance, we found significant negative correlations between SSS’ initial level and its growth rate, ST’s initial level and its growth rate, and SRH’s initial level and its growth rate. In addition, SSS indirectly affected the SRH of older adults through the initial level and growth rate of ST. Despite some limitations, this study raises some interesting questions about older adults’ health status in China, especially since China is in the midst of social transition, during which the family as a social subsystem has undergone drastic changes. These changes have affected older adults’ SSS to some extent and their health [102]. In addition, the outbreak of the COVID-19 epidemic in late December 2019 further highlighted the importance of targeted protection of the health and rights of older adults. Therefore, this study has important practical implications for improving the health of older adults and realizing active aging in China.

Combined with the results of data analysis, on the one hand, we should actively pay attention to vulnerable groups with low social status in older adults population, such as rural elderly, female elderly, elderly living alone (empty nesters), older adults with poor health status, etc. [103]. Furthermore, the government should establish a family-centered, community-supported social support system, focusing on material support, emotional communication, and the transfer of knowledge and skills for COVID-19 prevention and control for these groups [104]. On the other hand, in terms of improving the ST of older adults, we should build a friendly community environment [105] and organize a variety of social, cultural, and recreational activities to encourage older adults to interact with friends and neighbors, so as to further lift their ST level, reduce their sense of social isolation, and thereby improve their health [106].

## Data Availability

The datasets used and analyzed during the current study are available at www.isss.pku.edu.cn/cfps/en/.

## Abbreviations

SSS: Subjective social status
SRH: Self-rated health
ST: Social trust
CFPS: China Family Panel Studies
CPC: Communist Party of China
ISEI: International Socioeconomic Index
AHI: Annual household income
LGM: Latent growth modeling/model
SD: Standard deviation
SE: Standard error
CR: Critical ratio
